# Orally delivered rutin in lipid-based nano-formulation exerts strong antithrombotic effects by protein disulfide isomerase inhibition

**DOI:** 10.1080/10717544.2022.2083726

**Published:** 2022-06-08

**Authors:** Dan Chen, Yurong Liu, Peiwen Liu, Yang Zhou, Longguang Jiang, Cai Yuan, Mingdong Huang

**Affiliations:** a College of Chemistry, National & Local Joint Biomedical Engineering Research Center on Photodynamic Technologies, Fuzhou University, Fuzhou, China; b College of Biological Science and Engineering, Fuzhou University, Fuzhou, China

**Keywords:** Rutin, protein disulfide isomerase, self-nanoemulsifying drug delivery system, antithrombotic drug, microcirculation

## Abstract

Thrombosis occurs in both macrovasculature and microvasculature, causing various cardio-cerebral vascular diseases. The lack of effective and safe antithrombotic drugs leads to a public health crisis. Mounting evidence suggests that protein disulfide isomerase (PDI) plays a critical role in the initial stage of thrombus formation, motivating the research of the feasibility of PDI inhibitors as novel anti-thrombotics. Rutin, one of the most potent PDI inhibitors, was reported to suppress platelet aggregation and thrombosis in animal models, but further studies and clinical translation were restricted due to its low aqueous solubility and oral bioavailability. In this work, we fabricated rutin-loaded lipid-based nano-formulation (NanoR) and characterized their physical–chemical properties, release profiles, pharmacokinetic process, and pharmacodynamic function against thrombosis in macrovessels and microvessels. NanoR provided increased solubility and dissolution of rutin to achieve earlier *T*_max_ and higher *C*_max_ than the sodium salt of rutin (NaR) after oral gavage. *Ex vivo* studies demonstrated that NanoR significantly inhibited thrombin generation and clot formation in the plasma of mice. Importantly, such effect was reversed by exogenous recombinant PDI, demonstrating the specificity of the NanoR. In direct current-induced arterial thrombosis model and ferric chloride-induced microvascular thrombosis model, NanoR exhibited greatly enhanced antithrombotic activity compared with NaR. NanoR also showed good safety performance according to tail bleeding assay, global coagulation tests, and histological analysis. Overall, our current results indicated that NanoR offers a promising antithrombotic treatment with potential for clinical translation.

## Introduction

Thrombosis, the occlusion of blood vessels by localized clots, can occur in both macrocirculation and microcirculation, and becomes the central pathological event in various vascular diseases, including myocardial infarction, stroke, and pulmonary embolism (Mackman, 2008; Wendelboe & Raskob, 2016). During clot formation, the recruitment of activated platelets occurs in parallel with the blood coagulation cascade. Both processes communicate and promote each other to generate glue-like fibrin to seal the injured vessel. Currently, antiplatelets and anticoagulants are a mainstay for thrombosis prevention. However, there is an inherent risk of bleeding for these agents, which results in adverse cardiovascular events and negates the potential clinical benefit (Mackman, 2008; Melnikova, 2009; Lin et al., 2015b). Therefore, there are extensive and continuous efforts to develop novel antithrombotics with favorable potency and safety.

A flavonoid, quercetin-3-*O*-rutinoside or rutin, was identified to block thrombus formation in the mouse model and reduce thrombin generation in humans through targeting the extracellular protein disulfide isomerase (PDI) (Jasuja et al., 2012; Stopa et al., 2017; Zwicker et al., 2019). PDI is a thiol isomerase that catalyzes disulfide formation, reduction, and isomerization. The extracellular PDI plays an important role in the initiation of clot formation by regulating the oxidation states of labile disulfide bonds in critical hemostatic proteins, including platelet surface receptors αIIbβ3 and GPIbα, adhesive proteins TSP-1 and vitronectin, and coagulation factors (Versteeg & Ruf, 2007; Cho et al., 2008; Reinhardt et al., 2008; Furie & Flaumenhaft, 2014; Liang et al., 2021). The inhibitory potency of rutin on PDI was shown to be at the micromolar range (Jasuja et al., 2012; Lin et al., 2015a). We have recently identified the molecule binding site of rutin on PDI using combined structural biology and mutagenesis techniques, and demonstrated that residue H256 in PDI is the key mediating the rutin inhibition on PDI (Liao et al., 2022). However, rutin has a low water solubility and limited membrane permeability, leading to incomplete absorption and poor oral bioavailability. Therefore, rutin is often administered in high doses to achieve antithrombotic effects, reducing patient compliance and restricting its clinical translation (Mauludin et al., 2009; Gullon et al., 2017; Pan et al., 2019).

Much effort has been devoted to improve the bioavailability of rutin over the past few decades. Troxerutin, a trihydroxyethylated derivative of rutin, was synthesized and intravenously injected to treat chronic venous insufficiency diseases in China (Panat et al., 2016). However, serious side effects of troxerutin are reported in clinical practice. Salt formation is another commonly applied approach to increase the solubility and dissolution rate of acidic and basic drugs. To achieve this goal, careful attention must be paid to selecting and identifying the optimal salt forms for the drug, which is still a trial and error process (Serajuddin, 2007). Meanwhile, nanotechnology engineering holds great potential to overcome the obstacles in drug delivery, including polymeric nanoparticles, liposomes, dendrimers, lipid-based formulations, and nanocrystals (Machado et al., 2021). Lipid-based nano-formulations, such as self-nanoemulsifying drug delivery system (SNEDDS), have been extensively explored as an efficient approach to improve the dissolution and oral absorption of drugs with low water solubility and poor permeability (Porter et al., 2008). SNEDDS, a homogeneous mixture of the drug, oils, surfactants, and co-surfactants, spontaneously disperses in gastrointestinal fluids and forms nanoemulsion under gentle peristalsis (Date et al., 2010). In addition, the small droplet size and deformability makes SNEDDS capable of penetrating mucus and overcoming the absorption barrier of epithelial lining (Griesser et al., 2018). Therefore, this kind of lipid-based nano-formulation could be a promising strategy to address the drawbacks of natural rutin used as an oral antithrombotic agent.

Herein, we developed rutin-loaded SNEDDS (NanoR) with ultra-small size, homogeneous size distribution, and nearly charge-neutral surface. *In vitro* dissolution of rutin and oral absorption of NanoR were studied. *Ex vivo* antithrombotic effect of NanoR was evaluated using thrombin generation assay (TGA) and clot formation assay. Next, we established both macrovascular and microvascular thrombosis models, i.e. direct current-induced arterial thrombosis in the common carotid artery (CCA) and ferric chloride-induced microvascular thrombosis in the dorsal skinfold chamber (DSFC) model, and demonstrated that NanoR remarkably inhibited *in vivo* thrombus formation.

## Materials and methods

### Materials

Rutin (purity 98%) was purchased from J&K Scientific (Beijing, China). Caprylocaproyl macragol-8 glycerides (Labrasol) and Cremophor EL were purchased from Bioduly Biochemical Reagent (Nanjing, China). PEG-40 hydrogenated castor oil (RH 40) was purchased from Macklin Biochemical Co., Ltd. (Shanghai, China). Isopropyl myristate, glyceryl triacetate, and oleic acid were purchased from Aladdin Reagent Co., Ltd. (Shanghai, China). Gly-Pro-Arg-Pro peptide was synthesized and purified by GL Biochem Ltd. (Shanghai, China). Human thrombin was purchased from Hematologic Technologies Inc. (Essex Junction, VT). Thrombin chromogenic substrate S2238 was from Chromagenix (Bedford, MA). All the other chemicals were of analytical grade and purchased from Shanghai Chemicals Inc. (Shanghai, China).

### Animals

Healthy male Sprague-Dawley (SD) rats weighing 250–300 g and male ICR mice weighing 25–28 g were purchased from the Experimental Animal Center of Fuzhou (Fuzhou, China) and housed in an air-conditioned room at 25 ± 2 °C, in a 12 h light–dark cycle, with free access to food and water. Experimental protocols and handling of the animals were conducted in accordance with the guidelines for control and supervision of laboratory animals as approved by the institute animal ethics committee in Fuzhou University.

### Design and preparation of lipid-based nano-formulation for rutin (NanoR)

First, we measured the saturation solubilities of rutin in various surfactants (Labrasol, Tween-80, RH 40, and Cremophor EL), co-surfactants (PEG 400, 1,2-propylene glycol, and ethanol), and oils (glyceryl triacetate, isopropyl myristate, and oleic acid) according to shake flask method (Baka et al., 2008). Briefly, the excess amount of rutin was respectively added into 1 mL of each of the selected excipients mentioned above. After shaking at 37 °C for 72 h to reach equilibrium, it was centrifuged at 8000 rpm for 15 min and the supernatant was diluted with methanol as necessary. The concentration of rutin dissolved in each excipient was determined by UV/VIS spectrophotometer at 360 nm using methanol as a blank. The calibration curves were linear in the range from 3.12 to 100.0 μg/mL (*R*^2^=0.9993 in methanol). Each measurement was repeated in triplicate.

Next, the selection of the proper excipients was based on the solubility of rutin and self-emulsifying efficiency. A range of self-emulsifying formulations were prepared by mixing different ratios of components. Then, 50 μL of each formulation was added dropwise into 10 mL of double distilled water under mild stirring for 3 min at room temperature. The self-emulsifying efficiency of these formulations was assessed by visual observation (Kazi et al., 2019). Those with clear or translucent appearance were identified as the formation of nanoemulsions. The droplet size and polydispersity index (PDI) of the diluted systems were measured by Zetasizer Nano-ZS (Malvern, Worcestershire, UK) with disposable polystyrene cells at a backscatter angle of 173° at 25 °C. The solubility of rutin in the formulations was also determined using the shake flask method mentioned above. The optimized formulation was then identified, which has high self-emulsifying efficiency, uniformly dispersed small droplets and high maximum drug loading capacity. In addition, surface charge of the optimized formulation was determined using the technique of laser Doppler electrophoresis in combination with phase analysis light scattering by Nano-ZS at 25 °C.

### *In vitro* release of rutin

To investigate the effects of lipid-based nano-formulation on rutin release behavior, *in vitro* release study was performed using a bulk-equilibrium reverse dialysis method at 37 °C. In this method, the lipid-based nano-formulation was directly added into the release medium where it was able to release the loaded drug under maximum dilution. The nanodroplets were directly exposed to the external medium under perfect sink condition, which reflected a biological environment (Levy & Benita, 1990). Rutin was also prepared into salt formation (NaR) by dissolving in 0.1 M NaOH solution and adjusting the pH to 9 with 1 M HCl (Pan et al., 2019). Then, NaR was used as a control in the *in vitro* release study and the following studies. The dissolution media used were simulated gastric fluid (SGF, 0.1 M HCl) and simulated intestinal fluid (SIF, phosphate buffer solutions with pH 6.8), all of which contained 2.5% Tween 80 (Xu et al., 2012). Empty and ligated dialysis sacs (cellulose membrane, molecular weight cutoff 14 kDa, Sigma-Aldrich, St. Louis, MO) were pre-equilibrated with each medium for about 1 h prior to dialysis experimentation. One milliliter of NanoR or NaR was added into 900 mL of each release medium (outside the dialysis bags). The release medium inside the dialysis bags represents the receiver phase. At 30-min intervals during a 6-h period, one dialysis sac was taken out, 2 mL of aliquot was withdrawn from the interior of the dialysis sac, and an equal volume of fresh media was added into the medium. Rutin concentration was measured using UV/VIS spectrophotometer at 360 nm. The calibration curves were linear in the range from 4.95 to 49.5 μg/mL (*R*^2^=0.9999 in 0.1 M HCl, *R*^2^=0.9997 in PBS with pH 6.8). Each measurement was repeated in triplicate.

### Pharmacokinetic study

SD rats were randomly divided into two groups (*n* = 3) and fasted for 12 h before the experiment with free access to water. The bioavailability of NanoR was compared with that of NaR. Both NanoR and NaR were orally administered at a dose of 50 mg/kg. 0.5 mL of blood sample was collected from the retro-orbital venous plexus of each rat at 0.5, 1, 2, 3, and 4 h after oral gavage. The rats were provided with normal diet after 1 h of dosing. Plasma was separated by centrifuging the blood samples at 4000 rpm for 10 min and stored at −80 °C until analysis.

Extraction of rutin from the plasma samples was conducted by liquid–liquid extraction procedure. Briefly, protein precipitation was carried out by adding 200 μL of acetonitrile solution containing 1% trifluoroacetic acid (TFA) to 100 μL of plasma samples. The samples were vortexed for 3 min, incubated at 4 °C for 30 min, and centrifuged at 8000 rpm for 15 min. The organic layer was then transferred to a clean tube, completely dried under nitrogen gas at 40 °C, and reconstituted with HPLC mobile phase.

HPLC analysis was performed with a SinoChrom ODS-BD C18 column (4.6 × 150 mm, 5 μm). The mobile phases were water (A) and acetonitrile (B), both containing 0.1% TFA. The gradient elution included an initial wash using 12% B for 5 min at a flow rate of 0.4 mL/min, linearly increased to 100% B in 25 min at a flow rate of 1 mL/min, maintained at 100% B for 5 min at a flow rate of 1 mL/min, and then changed to the initial composition (12% B) in 1 min, followed by an equilibrium period of 10 min. The injection volume was 20 μL. UV detection was performed at 368 nm. The retention time was 14.5 min for rutin, and the lowest detection limit was 0.09 μg/mL. A linear calibration curve in the concentration range of 0.09–11.52 μg/mL was used to quantify rutin.

### Thrombin generation assay

SD rats were randomly divided into three groups (*n* = 6-8). Whole blood was collected via the ocular vein of rats before (baseline) and 2 h after oral gavage of NaR or NanoR (50 mg/kg), transferred into sodium citrate tubes, and centrifuged at 1200×*g* for 10 min to isolate plasma. Plasma was diluted with Tris–HCl buffer (20 mM Tris–HCl pH 7.4, 150 mM NaCl, 1 mM DTT, 1 mM EDTA, and 5% glycerin) in the presence of 5 mM Gly-Pro-Arg-Pro peptide and stimulated with 0.1 U/mL thrombin and 6 mM CaCl_2_. For rutin neutralization study, 48 μM PDI was added. Plasma samples were incubated for 90 minutes at 37 °C with rotation and diluted into PBS (1:20, v/v) containing 100 μM chromogenic substrate S2238 and 12.5 mM EDTA. Thrombin levels were detected by optical absorbance at 405 nm at 37 °C for 30 min using microplate reader (Spectra Max i3x, Molecular Devices, San Jose, CA). The initial velocities of the reaction were calculated based on the linear fitting of the O.D. values of the initial 10 minutes.

### Clot formation assay

SD rats were grouped (*n* = 5) and intragastrically administered with saline, NaR, or NanoR (50 mg/kg). After 2 h, plasma samples were obtained as mentioned above. Clot formation was triggered by adding Ca^2+^ (10 mM final concentration) into 30% plasma in a buffer containing 50 mM Tris–HCl and 150 mM NaCl (pH 7.4) and monitored by optical absorbance at 405 nm at 37 °C for 40 min. To study the specificity of rutin for PDI inhibition, 48 μM exogenous recombinant PDI was added and incubated with plasma before the addition of Ca^2+^.

### Direct current-induced arterial thrombosis in common carotid artery

ICR mice were randomly divided into six groups (*n* = 8) and orally administered with saline, vehicle, NaR, and NanoR at doses of 25 mg/kg and 50 mg/kg. After 2 h, mice were anesthetized with 1.5% sodium pentobarbital (30 mg/kg). The left CCA was exposed and stimulated with 0.5 mA direct current on an animal thrombus formation instrument (YLS-14B, Ji’nan Yiyan Technology Development Co., Ltd., Ji’nan, China), leading to the gradual formation of mixed thrombus in CCA. The occlusive rate (%) of CCA was monitored every 4 s by a far-infrared detector. The time when the occlusive rate reached 95% (time to occlusion) was recorded.

### Ferric chloride-induced microvascular thrombosis in dorsal skinfold chamber model

ICR mice were randomly divided into three groups (*n* = 6) and orally administered with saline, NaR, and NanoR at a dose of 50 mg/kg. At 1.5 h post-dosing, mice were anesthetized with 1.5% sodium pentobarbital (30 mg/kg). Dorsal skinfold of the mice was pulled out, and a titanium clipper with a central circular open window was clipped onto the skin and fixed by two screws. The exposed dermis and subcutis in the middle of the titanium clipper on one side of the skinfold were surgically removed and covered up with a circular coverslip. At 2 h post-dosing, the coverslip was removed, and 1 μL of 8% FeCl_3_ solution was applied to the surface of one selected microvessel of 40–60 μm in diameter, and the blood flow was monitored every 5 s using laser speckle contrast imaging for 10 min. The changes of relative blood flow velocity (rBFV) were shown as the percentage of the initiative rBFV.

### Tail bleeding and hemoglobin assay

Bleeding times and the volume of blood loss were determined as previously described (Liu et al., 2012). ICR mice were randomly assigned to receive saline, vehicle, or NanoR (25 mg/kg and 50 mg/kg) orally. After 2 h, the tails of mice were transected 0.5 cm from the tip and immediately immersed into a 15 mL tube containing 10 mL of pre-warmed saline. Bleeding time was recorded until the cessation of bleeding for 10 s. Blood loss was quantified by measuring the amount of hemoglobin in the saline. After centrifugation at 2000×*g* for 10 min, the pellet was collected and lysed with 2 mL lysis buffer (8.3 g/L NH_4_Cl, 1.0 g/L KHCO_3_, and 0.037 g/L EDTA). The absorbance at 575 nm was measured.

### Global coagulation tests

Blood was mixed with 3.2% sodium citrate (9:1, v/v). Plasma was prepared by centrifugation at 4000×*g* for 10 min to remove cellular elements. Global coagulation tests, including prothrombin time (PT) and activated partial prothrombin time (APTT), were detected using an automation coagulator analyzer (CA-660, Sysmex, Kobe, Japan) following the standard protocol.

### Oral toxicity study

The oral toxicity of NanoR was studied. ICR mice were randomly divided as follows: control group, vehicle group, low dose NanoR (25 mg/kg/day), and high dose NanoR (50 mg/kg/day) containing five animals in each group. At the end of the 15-day continuous treatment period, the organs of each mouse were collected and fixed in 4% paraformaldehyde, including heart, lung, liver, kidney, and spleen. The fixed organs were embedded in paraffin, sliced into sections with a thickness of 6 μm, and stained with hematoxylin–eosin (H&E). The stained slides were photographed using a light microscope (Leica MC170, Wetzlar, Germany).

### Statistics

All data were expressed as mean ± SD. Student’s *t*-test, one-way ANOVA followed by Tukey’s multiple comparisons test, and two-way ANOVA followed by Dunnett’s multiple comparisons test were performed, and the differences between groups were considered statistically significant if *p* < .05.

## Results

### Design, preparation, and characterization of NanoR

We firstly measured the saturation solubilities of rutin in various excipients (Figure S1). Rutin exhibited highest solubility in 1,2-propylene glycol (32.0 ± 2.0 mg/mL), so that it was chosen as the co-emulsifier in the following formulations. Among the surfactants screened, Labrasol had the greatest solubilizing potential of rutin (5.2 ± 0.18 mg/mL), and was selected as an emulsifier. The solubilities of rutin in the three selected oils (isopropyl myristate, oleic acid, and glyceryl triacetate) were much lower than those in co-surfactants and surfactants, showing limited solubilization property. Next, we preset the ratio of Labrasol and 1,2-propylene glycol at 3:1 (S/CoS), and examined various combinations of oils and S/CoS mixture to investigate their ability to form emulsions. The pictures in Figure S2 showed different transparencies of the formulations upon aqueous dispersion. The resulting emulsions with glyceryl triacetate as oil phase appeared bluish to transparent visually, demonstrating good efficiency of the selected S/CoS mixture to emulsify glyceryl triacetate. However, this S/CoS mixture emulsified isopropyl myristate and oleic acid poorly, as evidenced by the bluish to milky or turbid in appearance of the resulting emulsions. The result suggested that glyceryl triacetate could serve as the suitable oil phase in this self-emulsifying system. Yet, subsequent formulation optimization using different ratio of S/CoS mixture (2:1, 1:1, and 1:2) failed to improve the emulsification efficiency, as the droplet size of the resulting emulsions ranged from 250 to 800 nm with low monodispersity (PDI of 0.3–0.8, data not shown). Thus, RH 40 was also added in the formulation as an emulsifier. As shown in Figure S3(A), the emulsions gradually became clear as the ratio of RH40 to Labrasol increased (1:9 to 9:1) in the formulation with 50% glyceryl triacetate and 12.5% 1,2-propylene glycol, indicating the improved emulsification efficiency by RH 40. The ratio of RH 40 to Labrasol at 5:5 (*S*_mix_) in the system yielded a resulting emulsion with droplet size less than 200 nm (Figure S3B), which was used in the following formulations since further increase of the portion of RH 40 in the system would weaken the solubilization of rutin. Subsequently, 12 formulations were prepared, containing glyceryl triacetate as oil phase (in the percentage range of 10–30%), the combination of RH 40 and Labrasol at the selected *S*_mix_ as emulsifier and 1,2-propylene glycol as co-emulsifier (S/CoS = 3:1, 2:1,and 1:1) (Table S1). The results in Table S1 showed that F2, F5, and F7 with small globule size (less than 50 nm) and homogeneous dispersion were suitable formulations for subsequent rutin loading. The saturation solubility of rutin in F2, F5, and F7 was 24.3 ± 3.2 mg/mL, 19.2 ± 2.8 mg/mL, and 16.2 ± 3.7 mg/mL, respectively. These results indicated that F2 had the highest drug loading capacity, and was the optimal formulation in our study.

**Figure 1.**
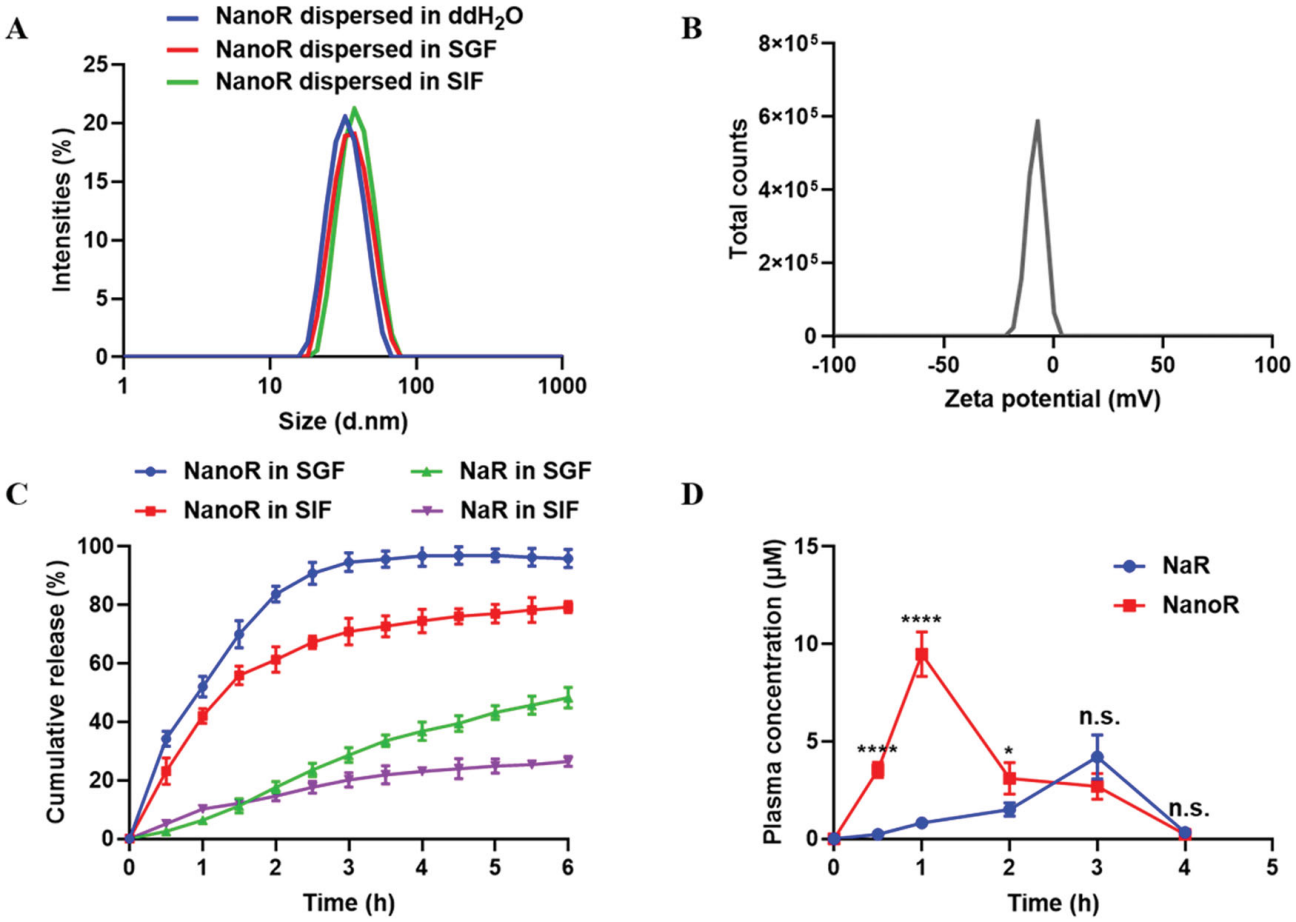
Characterization of NanoR. (A) Size distribution of NanoR dispersed in ddH_2_O, SGF, and SIF. (B) Zeta potential of NanoR dispersed in ddH_2_O. (C) *In vitro* cumulative release profile of NanoR and NaR in SGF and SIF, respectively. (D) Plasma concentrations of rutin delivered by lipid-based nanoformulation or sodium formation after oral administration. *p < .05, ****p < .0001.

The optimized lipid-based nano-formulation with 10% glyceryl triacetate, 30% Labrasol, 30% RH 40, and 30% 1,2-propylene glycol gave a maximum drug loading capacity of 24.3 ± 3.2 mg/mL, which was about 194-fold of the saturation solubility of rutin in water (0.125 mg/mL). Thus, NanoR with a drug loading efficiency of 20 mg/mL was prepared and used in the following studies. Upon diluting NanoR in aqueous media, a transparent dispersion system with nanodroplets was formed, giving an average particle size of 42.6 ± 2.8 nm with a low polydispersity (0.15 ± 0.08). The particles carried slightly negative charges with a zeta potential of −4.2 ± 0.32 mV (Figure 1(A,B)). The result demonstrated that drug loading in lipid-based nano-formulation did not significantly influence the droplet size and surface charge of the emulsions upon aqueous dilution.

### NanoR exhibited fast dissolution *in vitro*

*In vitro* dissolution studies were evaluated using SGF (pH 1.2) and SIF (pH 6.8) under sink conditions. 2.5% Tween 80 in the dissolution buffer helped to keep sink condition, and did not affect the self-emulsification of the formulation, as the droplet size of NanoR upon dilution in the dissolution buffer was not significantly different from that in water (data not shown). As seen in Figure 1(C), in acidic media, the release of rutin from NanoR reached 34% within 0.5 h, and almost complete release (90.76 ± 3.78%) was achieved at 2.5 h. The media with a near neutral pH slowed down the release rate of rutin from nano-formulation, which reached approximately 80% of rutin at 6 h. NaR, the sodium salt of rutin dissolved in alkaline condition (pH 9.0), underwent fast precipitation followed by redissolution in SGF, and the cumulative release of NaR reached nearly half (48.32 ± 3.50%) at 6 h. The poor solubility of rutin in the medium at pH 6.8 led to the low dissolution rate of NaR, and the cumulative release at 6 h was only 26.44 ± 1.65%.

### NanoR exhibited enhanced oral absorption

The pharmacokinetic profiles of sodium salt and nano-formulation of rutin were studied in SD rats with a single dose of 50 mg/kg given by gavage. Bioanalytical method was developed and validated for measurement of rutin concentration in the rat plasma. The retention time of rutin was found to be 14.8 min with the absence of any interfering peaks derived from the plasma. The molar concentration of plasma rutin vs. time curves is displayed in Figure 1(D). It has been reported that rutin is subjected to extensive metabolism during oral absorption, including hydrolysis and glycosylation (Erlund et al., 2000). It is noteworthy that the prototype rutin with concentrations above the detection limit of our method (0.0485 μg/mL) was not detected in both groups administered with NaR or NanoR after 4 h. The rutin-loaded nano-formulation clearly showed faster and better oral absorption of rutin than NaR. The orally administered NanoR showed rapid absorption with a *T*_max_ of 1 h, whereas NaR exhibited a prolonged *T*_max_ of 3 h. *C*_max_ of rutin delivered by nano-formulation reached 9.48 ± 1.14 μM, which was significantly higher than that delivered in salt formation (4.20 ± 1.13 μM, *p*=.0006). Furthermore, the calculated AUC_0–4 h_ of NanoR exhibited significant 2.24-fold increase compared with NaR (*p* < .0001).

### NanoR inhibited thrombin generation and clot formation *ex vivo*

**Figure 2.**
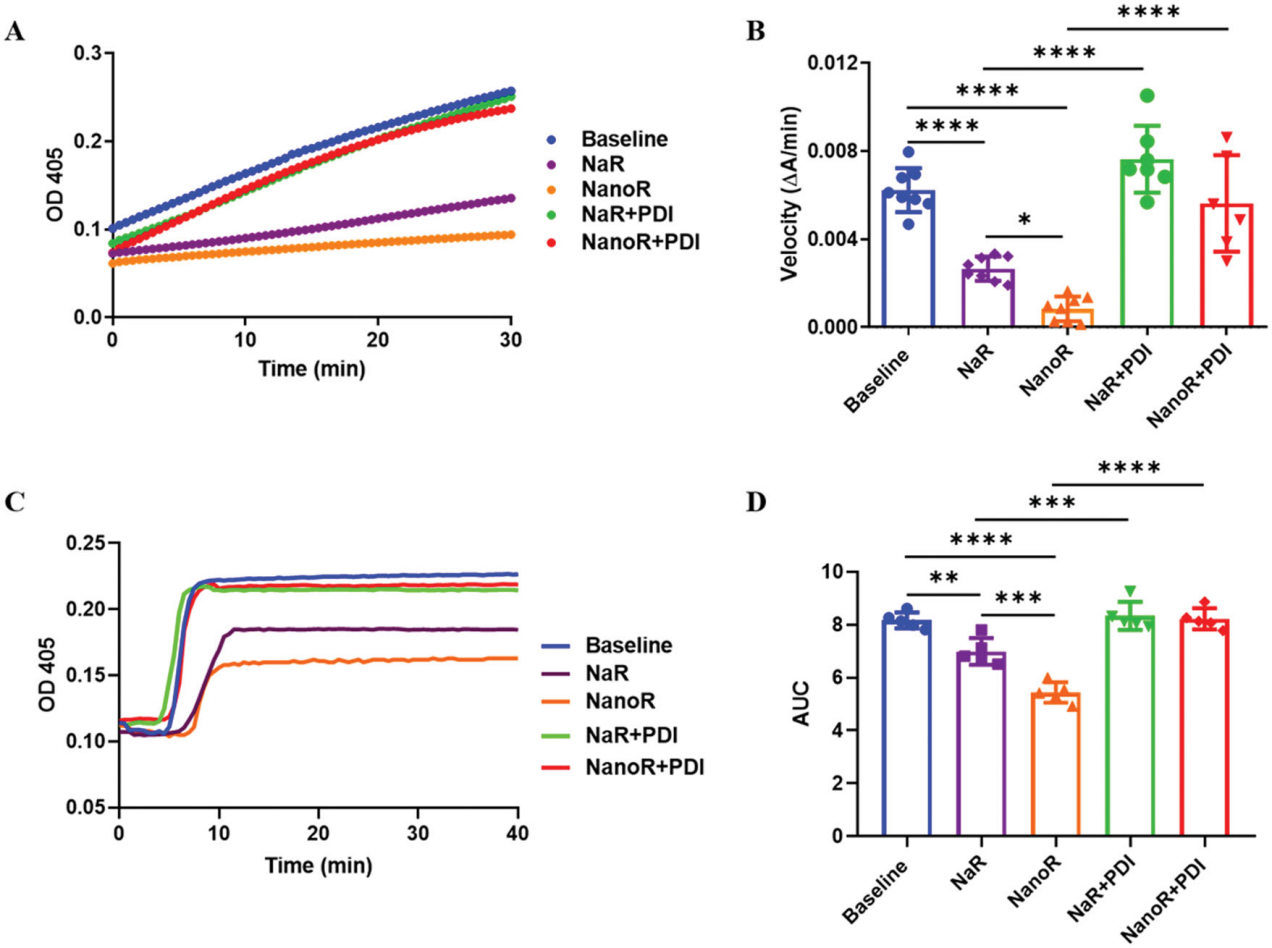
NanoR inhibited thrombin generation and clot formation *ex vivo*. (A) Thrombin generation in the plasma withdrawn from mice before (baseline) and 2 h after oral gavage of NanoR or NaR in the absence and presence of excessive exogenous PDI (48 μM). (B) The initial velocities of thrombin generation calculated based on the linear fitting of the O.D. values of the initial ten minutes in panel A. (C) Clot formation in the plasma withdrawn from mice before (baseline) and 2 h after oral gavage of NanoR or NaR in the absence and presence of excessive exogenous PDI (48 μM). (D) Area under the curve (AUC) of clot formation in panel C. **p < .01, ***p < .001, and ****p < .0001.

In order to evaluate the potential antithrombotic effects of NanoR and NaR, we first measured thrombin generation using a coagulation assay, where PDI activity plays a role in the initiation and burst of thrombin (Jurk et al., 2011). S2238, a short peptide that covalently links to 4-nitroaniline (pNA), is a thrombin-specific chromogenic substrate. The cleavage of pNA off the peptide by thrombin enables quantitative detection of thrombin by spectrophotometer at 405 nm. Following oral administration of NanoR or NaR, we observed a significant reduction of thrombin generation at 2 h compared with baseline (*p* < .0001), as revealed by the cleavage rate of the chromogenic substrate. Besides, treating with NanoR was even more effective in reducing thrombin generation when compared with NaR (*p* = .0435). To study the specificity of rutin inhibition on PDI, we added exogenous recombinant PDI (48 μM) onto the TGA buffer of the NanoR treated plasma. The results showed that exogenous PDI restored the thrombin generation back to the baseline level (Figure 2(A,B), baseline vs. NanoR + PDI: *p*> .05, NanoR vs. NanoR + PDI: *p* < .0001), indicating that exogenous recombinant PDI (48 μM) reversed the effect of NanoR on thrombin generation. Similarly, for the NaR gavage group, the addition of 48 μM recombinant PDI to the plasma restored thrombin generation at 2 h following NaR gavage (baseline vs. NaR + PDI: *p*> .05, NaR vs. NaR + PDI: *p* < .0001).

Next, we assessed the coagulative function of plasma samples in different groups via kinetic microplate-based clot formation assay with coagulation initiated by the addition of 10 mM calcium ion (Figure 2(C,D)). Fibrin formation and polymerization happened at about 5 min upon calcium addition, and stable clot was formed at about 8 min in control plasma. Treatment with NaR or NanoR postponed fibrin formation until 6–7 min. In addition, the clot size (the maximum absorptions of stable clots) dropped dramatically after NaR or NanoR ingestion. Quantitatively, the clot AUC (area under curve) was greatly decreased to 85.6% and 66.6% of the baseline in NaR- and NanoR-treated group, respectively (baseline vs. NaR: *p* = .003, baseline vs. NanoR: *p* < .0001). NanoR gavage produced a stronger anticoagulatory effect, as clot AUC in NanoR-treated group was significantly smaller than that in NaR-treated group (*p* = .0001). Exogenous PDI rescue was also performed in this assay. The result showed that clot formation was normalized following incubation with 48 μM recombinant PDI in both the NaR- and NanoR-treated groups (NaR vs. NaR + PDI: *p* = .0007, NanoR vs. NanoR + PDI: *p* < .0001), and no significant differences of clot AUC were observed among baseline, NaR + PDI and NanoR + PDI groups. These rescue results clearly demonstrated the specificity of Rutin to PDI, consistent with our previous studies showing the direct binding of rutin to PDI (Lin et al., 2015a; Wang et al., 2018).

### NanoR inhibited carotid artery thrombosis induced by electric current *in vivo*

**Figure 3.**
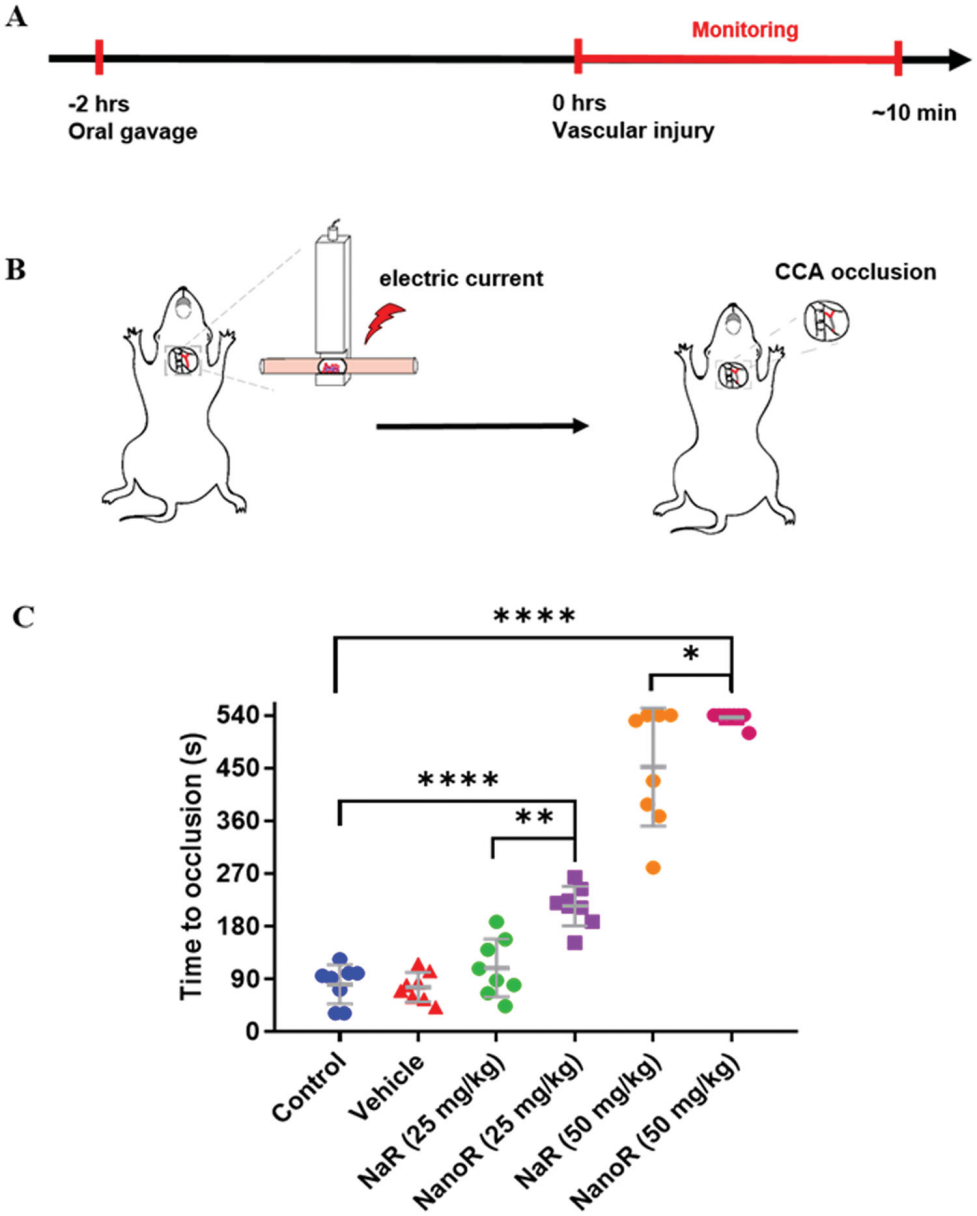
NanoR inhibited carotid artery thrombosis induced by electric current *in vivo*. (A) Timeline of antithrombotic therapy and common carotid artery (CCA) injury. (B) Schematic diagram of vascular injury induced by electric current and subsequent CCA occlusion. (C) Time to occlusion in the CCA of mice induced by electric current stimulation (0.5 mA) 2 h after oral administration with saline, vehicle (blank-SNEDDS), NaR and NanoR at doses of 25 mg/kg and 50 mg/kg, respectively. *p < .05, **p < .01, and ****p < .0001.

We used a murine model of direct current-induced arterial thrombosis in CCA to assess the antithrombotic effects of NanoR *in vivo*. Electric current can injure endothelia of CCA and induce thrombosis subsequently. The occlusion rate in the CCA was monitored in real-time. It is shown in Figure 3 that the time-to-occlusion (TTO) in the control group was 81.0 ± 33.4 s with electric current of 0.5 mA, and treating with vehicle (the lipid-based nano-formulation without rutin) did not significantly affect the TTO *in vivo*. TTO was prolonged in NaR- and NanoR-treated groups in a dose-dependent manner. In NaR-treated mice, TTO was prolonged to 109.0 ± 49.2 s at a dosage of 25 mg/kg, but no significant difference was observed compared with control. At doubled dose of NaR, the TTO was significantly prolonged to 451.9 ± 100.6 s (*p* < .0001 compared with control), with three out of eight mice did not even show complete artery occlusion in 9 min. Both low and high doses of NanoR notably inhibited thrombosis *in vivo* with the TTO of 214.8 ± 33.9 s and 536.2 ± 10.6 s, respectively (*p* < .0001 compared with control). Seven out of eight mice in NanoR-treated group with a dose of 50 mg/kg did not yield complete CCA occlusion in this experiment. Compared with NaR treatment, NanoR had a greater ability to inhibit thrombosis (*p* = .002 when treated with a dose of 25 mg/kg, *p* = .022 when treated with a dose of 50 mg/kg).

### NanoR inhibited microvascular thrombosis induced by FeCl_3_ in dorsal skinfold chamber model

**Figure 4.**
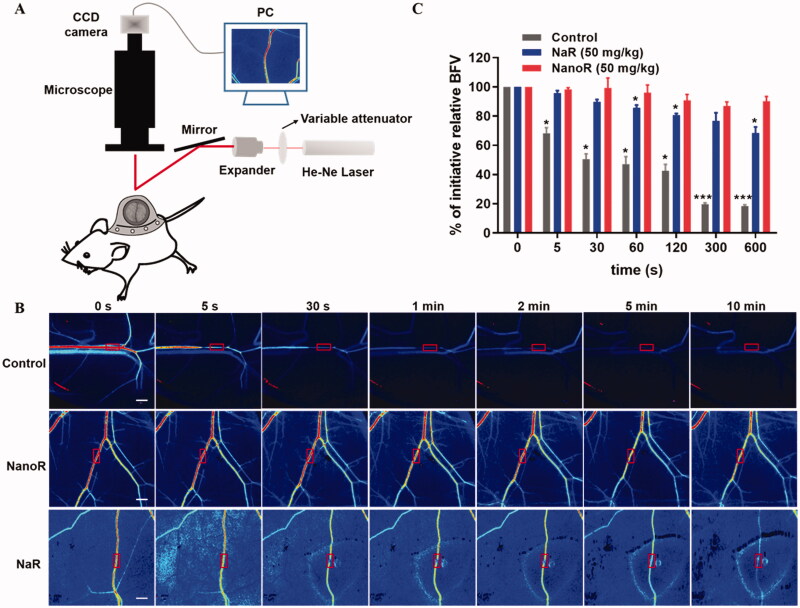
NanoR inhibited microvascular thrombosis induced by FeCl_3_ in dorsal skinfold chamber (DSFC) model. (A) Schematic of the laser speckle contrast imaging system and DSFC model. (B) Laser speckle imaging of changes in blood flow in ROIs (red rectangles) induced by FeCl_3_ 2 h after oral delivery of saline, NanoR and NaR (50 mg/kg), respectively. The ROIs also indicated where FeCl_3_ (8%, 1 μL) was applied. Scale bar: 0.5 mm. (C) Relative blood flow velocity (BFV) at pre-determined time points expressed as percent change from baseline (*t* = 0 s). *p < .05 and ***p < .001 compared with BFV at 0 s.

We also used the murine dorsal skinfold chamber (DSFC) model to determine the occurrence of subcutaneous microvascular thrombosis after topical application of FeCl_3_, which affords the advantages of minimally invasive operation and continuous monitoring of the same microvessel in real time. As shown in Figure 4, the blood flow of the injured vessel in the control group fell off quickly just 5 s after FeCl_3_ application (*p* = .0385 compared with that at 0 s). The microvessel became almost static with an rBFV below 20% at 300 s, and the hypoperfusion was maintained until 600 s (*p* = .0003 compared with that at 0 s). In NaR-treated group, the blood flow of the injured vessel gradually dropped, and rBFV declined to 68.5 ± 7.0% after incubation with FeCl_3_ for 600 s (*p* = .0423 compared with that at 0 s). However, treating with NanoR greatly inhibited thrombus formation induced by FeCl_3_. The rBFV maintained around 85–100% during the experiment (10 min), and no significant differences were found among the rBFVs at various time points in NanoR-treated group.

### NanoR did not affect normal hemostasis and had good biocompatibility

**Figure 5.**
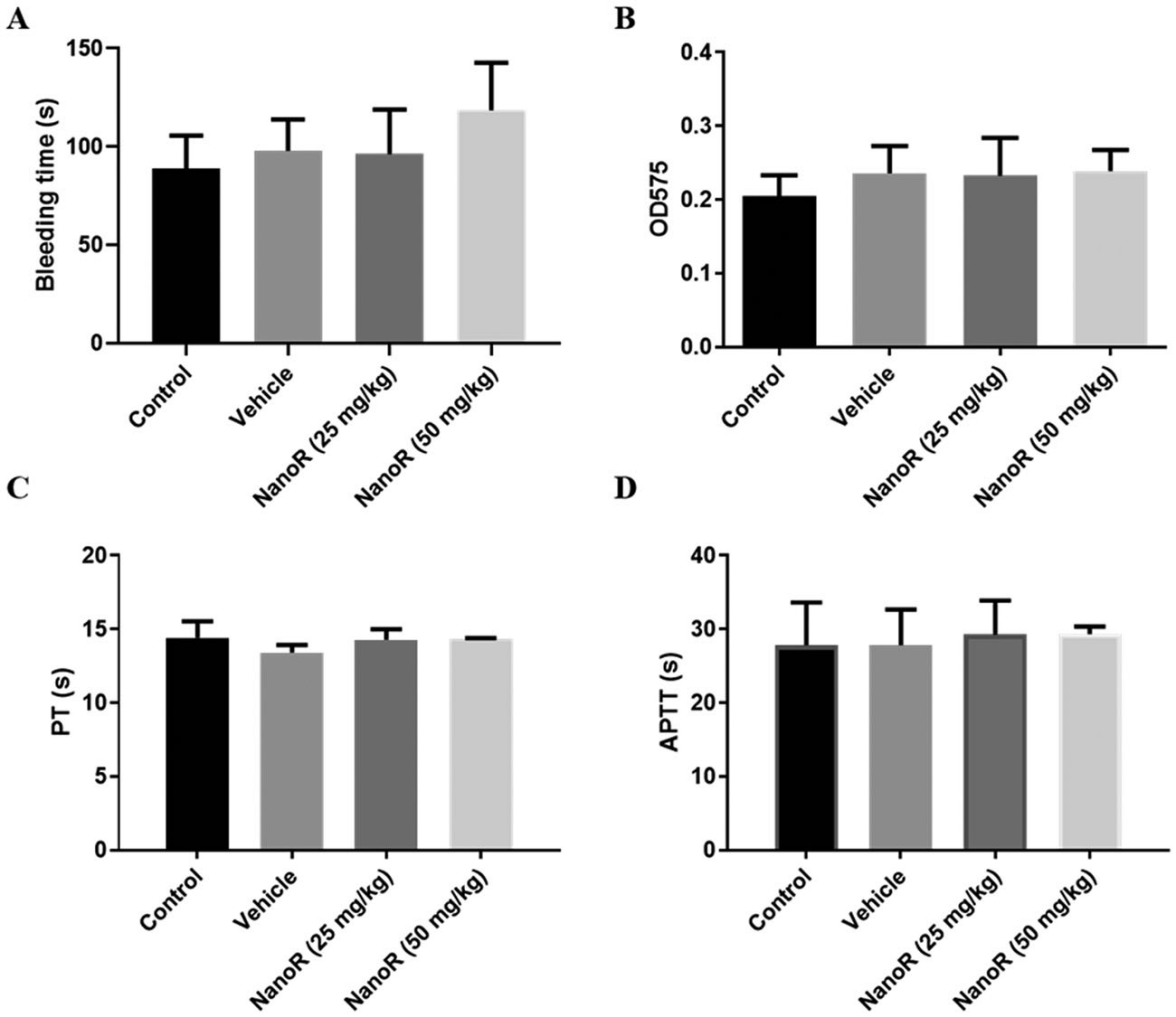
NanoR did not affect normal hemostasis. Tail bleeding time (A), hemoglobin loss (B), prothrombin time (C), and activated partial thromboplastin time (D) of normal mice treated with saline, vehicle (blank-SNEDDS) and NanoR at doses of 25 mg/kg and 50 mg/kg.

Next, we evaluated the effect of NanoR on the hemostasis of normal mice by tail bleeding assay. Mice in the control group had a bleeding time of 88.8 ± 16.7 s and a bleeding volume of 0.20 ± 0.03. NanoR with a dose of 50 mg/kg of slightly increased tail bleeding time without statistically significant difference compared with the control group. In mice receiving NanoR at the low and high doses (i.e. 25 mg/kg and 50 mg/kg), no significant differences were observed in hemoglobin loss compared with those in the control group (Figure 5(A,B)). Similarly, NanoR administration did not obviously affect the global coagulation in normal mice, as the PT and APTT in different groups remained almost unchanged (Figure 5(C,D)).

**Figure 6.**
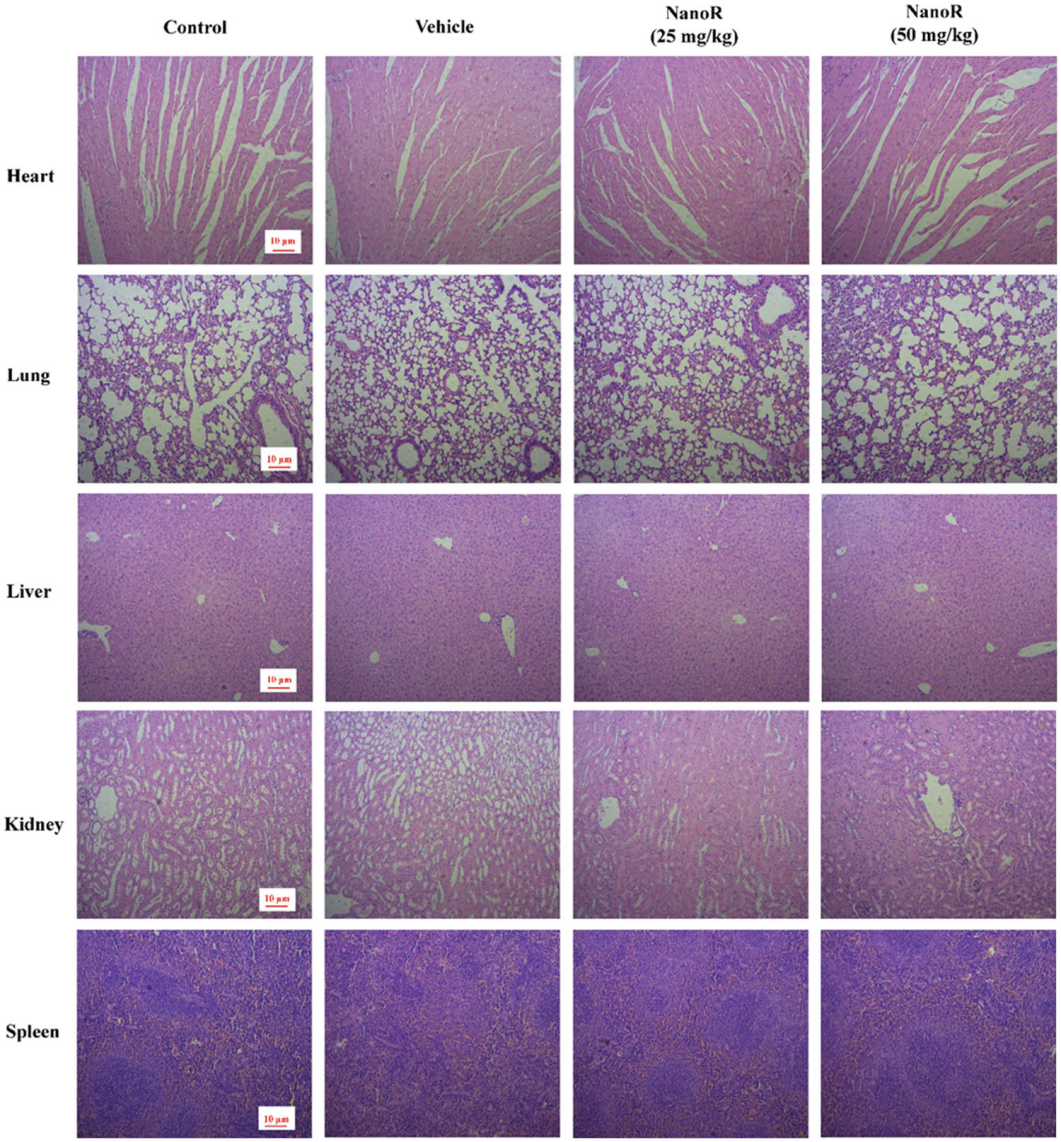
NanoR had good biocompatibility. Representative images of histological sections of major organs (heart, lung, liver, kidney, and spleen) withdrawn from mice after continuous dosing with saline, vehicle (blank-SNEDDS), and NanoR (25 mg/kg and 50 mg/kg) once a day for 15 days. Scar bar: 10 μm.

To further assess the *in vivo* biocompatibility, NanoR was orally administered to normal mice once a day for 14 days. Figure 6 shows the histopathology profiles of mice given NanoR, including heart, lung, liver, kidney, and spleen. No evident pathological differences or inflammatory cell infiltration were observed in the tissue section, indicating good *in vivo* biocompatibility of NanoR.

## Discussion

Impairment of coagulation/fibrinolysis balance, changes in blood rheology, and damages to endothelial lining cause thrombosis, leading to serious cardiovascular diseases gradually (Furie & Furie, 2008). Microvascular occlusion in arterioles, venules, and capillaries can occur in multiple tissue beds long before the onset of circulatory thrombosis, and can result in a partial or complete organ failure (Gutterman et al., 2016). Currently available antithrombotics include agents that directly inhibit platelet functions (antiplatelet therapy) and those that intervene in the coagulation cascade (anticoagulants). The anticoagulant market has been growing rapidly since the approval of direct oral anticoagulants (DOACs), i.e. direct thrombin inhibitors and direct FXa inhibitors (Verheugt et al., 2019). Apixaban and rivaroxaban, both of which are FXa inhibitors, account for two of the top 10 best-selling drugs worldwide in 2020. However, these drugs still confront prevalent underuse in clinical practice due to concerns with the increased risk of bleeding, especially in the elderly (Mackman et al., 2020). Moreover, no antidote for direct thrombin or FXa inhibitors is clinically approved to reverse the anticoagulants in bleeding events. In addition, the combination of antiplatelet and anticoagulant therapies is usually required in patients suffering from coexisting atrial fibrillation and other vascular diseases, e.g. stent thrombosis and coronary artery disease (Moser et al., 2014).

Recently, a surging number of researches have revealed that PDI plays a prominent role in the initial stage of thrombosis. In general, PDI contains endoplasmic reticulum (ER) retention signals and is found in high concentrations in the ER; however, PDI can also escape ER retention mechanism and localize to secretory granules and membrane surface in platelets and endothelial cells once vascular injury or inflammation occurs (Turano et al., 2002). However, a strong direct link between PDI and coagulation cascade or coagulation regulatory pathways has not been consensually established. What is encouraging is that some PDI inhibitors, e.g. quercetin and its derivatives, such as rutin, were found to have strong anticoagulatory effects without bleeding side effects (Jasuja et al., 2012). We showed that rutin binds directly to b’x domain of PDI, inducing a more compact conformation of PDI with less flexibility (Lin et al., 2015a). In clinical practice, troxerutin, a trihydroxyethylated derivative of rutin, has been proved to strengthen the wall of blood vessels, and is used as a vasoprotective (Wang et al., 2017).

In the present work, we developed a lipid-based nano-formulation for oral delivery of rutin in order to enhance its aqueous solubility and improve its bioavailability. The solubility and bioavailability of drugs have decisive roles in pharmacological actions *in vivo*. SNEDDS is one of the most well-developed and efficient lipid-based drug carriers. Large amounts of drug molecules are pre-dissolved in the excipients of the formulation, which ensures fast and increased oral drug absorption without the rate-limiting dissolution process. The NanoR we presented here had a drug load of 24.3 ± 3.2 mg/mL, and yielded an easily dispersible and stable nanoemulsions upon dilution in aqueous media. The emulsion droplets have a small size, narrow size distribution, and slightly negative charges, which ensure satisfactory mucosal permeation and improved absorption efficiency of loaded drugs. To date, some approaches have already been proposed for rutin oral delivery, such as sodium salt formation, nanocapsules, nanocrystals, cyclodextrin complex, phospholipid complex, and nanoemulsions (Sharma et al., 2013). Among these systems, a sodium salt of rutin (NaR) has been reported to increase its water solubility by at least 200-fold. Thus, we adopted this sodium salt formation of rutin as a control group in our study (Pan et al., 2019). The near complete release of rutin from NanoR indicated that rutin remained solubilized in simulated gastric and intestinal fluids, and available for further gastrointestinal absorption. In comparison, NaR showed limited release due to substantial precipitation in both acidic and neutral media, e.g. the GI tract. Besides, the presence of large amounts of NaCl in the GI environment also substantially influences the solubility and dissolution rate of salts via common-ion effect (Serajuddin, 2007). Serajuddin et al. reported a significant decrease in the solubility of the sodium salt of an acidic drug from 7.8 to 1.1 mg/mL after adding 0.1 M NaCl. Since the dissolution rate depends on the solubility of the drug in the diffusion layer in the vicinity of the drug particles, the influence of common-ion effect on the saturation solubility will also impact the dissolution rate (Serajuddin et al., 1987). *In vivo* pharmacokinetic study demonstrated an earlier and better absorption of rutin from NanoR than NaR, which further verified the improved solubility and immediate release of rutin in gastrointestinal media delivered by the lipid-based nanocarrier. Ramaswamy et al. reported that the *C*_max_ of rutin was 1.11 μg/mL delivered in chitosan nanoparticles after oral administration at a dose of 35 mg to rabbits weighing between 1.5 and 2 kg (Ramaswamy et al., 2017). After dose conversion, the *C*_max_ of rutin in NanoR (5.78 μg/mL) was about 3.4–4.5-fold of that in chitosan nanoparticles. Labrasol, one of the main components in the formulation, is a surfactant with a medium length alkyl chain. It has been reported that Labrasol is able to increase membrane lipid fluidity, which is beneficial to the drugs with poor membrane permeability (Jannin et al., 2008). Moreover, the production and scale-up process of SNEDDS is very simple and cost-efficient, providing an accessible approach to clinical translation.

We tested the antithrombotic effects of NanoR and NaR *ex vivo* and *in vivo.* Thrombin plays a pivotal role in the coagulation process, and its amount reflects the hemostatic function (Mann, 2003). Our *in vitro* study showed that native rutin with a concentration up to 50 μM did not affect the activity of thrombin (Figure S4). TGA results demonstrated significant inhibition of thrombin generation in the plasma withdrawn from rats 2 h after oral administration of NanoR and NaR. The addition of excess recombinant PDI to the post-treated plasma reversed the inhibition effect of rutin in TGA, which is strong support for the specificity of rutin for PDI inhibition. NanoR remarkably improved the effect of rutin on PDI inhibition, resulting in a more significant reduction in thrombin generation. In our clot formation study, the treatment with NanoR and NaR led to delayed clotting initiation time and a limited extent of clot formation. Again, NanoR exerted a stronger anticoagulatory effect than NaR, and excess exogenous PDI restored the coagulation function of pretreated plasma in clot formation assay. Recent studies revealed the importance of microcirculatory health in cardiovascular disease management (Struijker-Boudier, 2007). The link between obstructive atherosclerosis in the epicardial coronary arteries and myocardial ischemia is well established. In the past several decades, however, a number of studies have discovered that coronary microvascular dysfunction and remodeling contribute to myocardial ischemia in patients with normal coronary arteries. This finding has led to a growing consensus that microangiopathy can serve as an early culprit in the pathophysiology of heart disease (Camici & Crea, 2007; Labazi & Trask, 2017). It was also demonstrated that hypertension-dependent microvascular alterations contributed to the pathologic changes in the macrovasculature and subsequently to end-organ damage (Cohuet & Struijker-Boudier, 2006). In this study, we employed both microvascular and macrovascular thrombosis models to validate the antithrombotic potentials of nanoencapsulated rutin. Ferric chloride is extensively used to induce endothelial denudation and thrombosis via generating reactive oxygen species. Recently, a novel two-stage mechanism for the action of FeCl_3_ in thrombus formation has been proposed. The first stage is based on colloidal chemistry principles whereby cells and plasma proteins adhere and aggregate due to the binding of negatively charged proteins to positively charged iron species. The extent of this stage is dependent on effective FeCl_3_ concentration, which is governed by intraluminal mass transport. In the second stage, clot formation proceeds via platelet aggregation and activation of the coagulation cascade in response to both endothelial cell death and arrested blood components. Tissue factors and platelets have been found to adhere to the surface of ferric ion-filled spherical bodies, triggering platelet aggregation and thrombin generation (Ciciliano et al., 2015). Stimulation with ferric chloride was thus applied in combination with DSFC model to study the early stage of embolic thrombosis in microvasculature with a diameter of 40–60 μm. In the macrovascular thrombosis model, we used a physical method, direct current stimulation, to generate mixed clots, imitating the early thrombus in human. We chose the dose of 50 mg/kg in animal experiments based on the IC_50_ of rutin in PDI inhibition and the pharmacokinetic profile after NanoR oral administration. According to the insulin-based turbidimetric assay, rutin inhibits PDI in a dose-dependent manner with an IC_50_ of 6.1 μM (1.1–10.7 μM, 95% CI) (Jasuja et al., 2012). In our study, the plasma concentrations of rutin after NanoR oral administration in the first 3 h were within this range. In addition, rutin can bind to the blood vessel wall, which maintains antithrombotic activity but cannot be detected in plasma. Thus, it is rational to assume that oral delivery of NanoR with a rutin dose of 50 mg/kg is sufficient to exert a strong antithrombotic effect via PDI inhibition. *In vivo* results verified our deduction, and were consistent with the results of *in vitro* release profile, oral absorption and *ex vivo* pharmacological analysis. While the enhanced antithrombotic activity of rutin delivered by the lipid-based nano-formulation was achieved, no increased bleeding risk and pathological changes of major organs were observed according to the safety evaluation results.

## Conclusions

In summary, rutin-loaded SNEDDS was successfully prepared and characterized for the prevention of thrombosis both in large blood vessels and in microvasculature. NanoR had a small size, narrow size distribution, and near-neutral surface charge. NanoR augmented the solubility of rutin in gastrointestinal fluids, ensuring timely release of rutin for subsequent absorption. NanoR exhibited faster and better oral absorption of rutin compared with sodium salt of rutin. Administration with NanoR greatly inhibited thrombin generation and clot formation *ex vivo*. Furthermore, NanoR significantly delayed the TTO in a dose-dependent manner in the direct current-induced arterial thrombosis model and greatly preserved the blood flow in the ferric chloride-induced microvascular thrombosis model. Together, we provided an oral rutin delivery strategy based on a lipid-based nano-formulation with increased solubility, improved bioavailability, enhanced antithrombotic effect, and good safety profile, which holds great promise for clinical translation.

## Supplementary Material

Supplemental Material
