# Supplementary material for: Orally delivered rutin in lipid-based nano-formulation exerts strong antithrombotic effects by protein disulfide isomerase inhibition
Source: Drug Deliv. 2022 Jun 8;29(1):1824–35. doi: 10.1080/10717544.2022.2083726 (PMC9186361; doi:10.1080/10717544.2022.2083726)
Supplement: Supplemental Material [file IDRD_A_2083726_SM3365.docx]

Supporting Information

**For**

**Orally delivered rutin in lipid-based nano-formulation exerts strong antithrombotic effects by protein disulfide isomerase inhibition**

Dan Chen^a^, Yurong Liu^a^, Peiwen Liu^a^, Yang Zhou^a^, Longguang Jiang^a^, Cai Yuan^b,*^, Mingdong Huang^a,*^


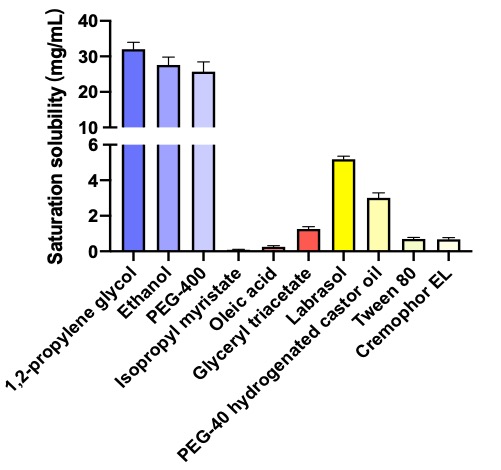


**Figure S1. The saturation solubilities of rutin in various cosurfactants (blue), oils (red) and surfactants (yellow).** Data are expressed as mean ± SD (n = 5).


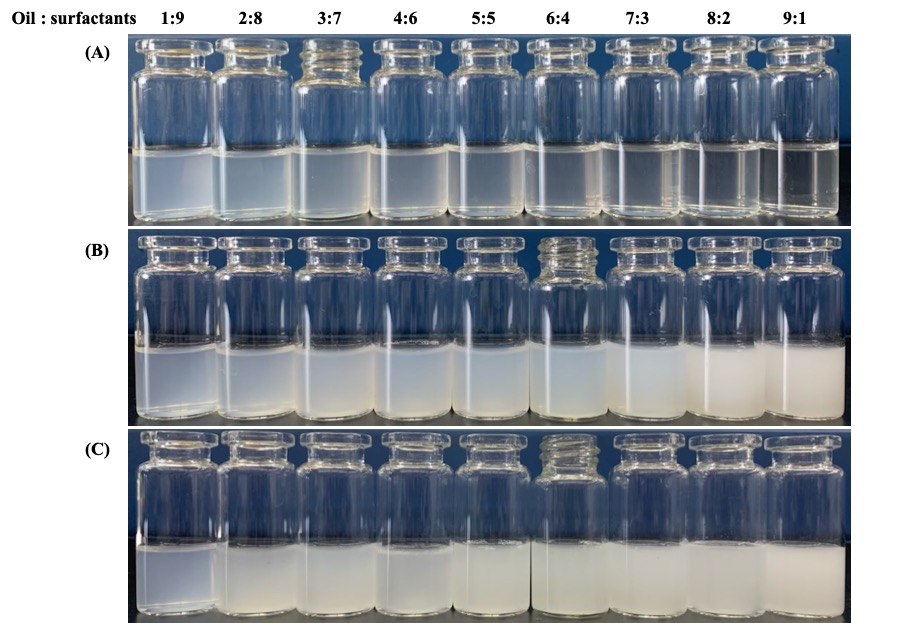


**Figure S2. Selection of oil for the lipid-based formulation.** Emulsification efficiency of Labrasol and 1,2-propylene glycol (at a ratio of 3:1) using different ratios of (A) glyceryl triacetate, (B) isopropyl myristate and (C) oleic acid as oily phase.


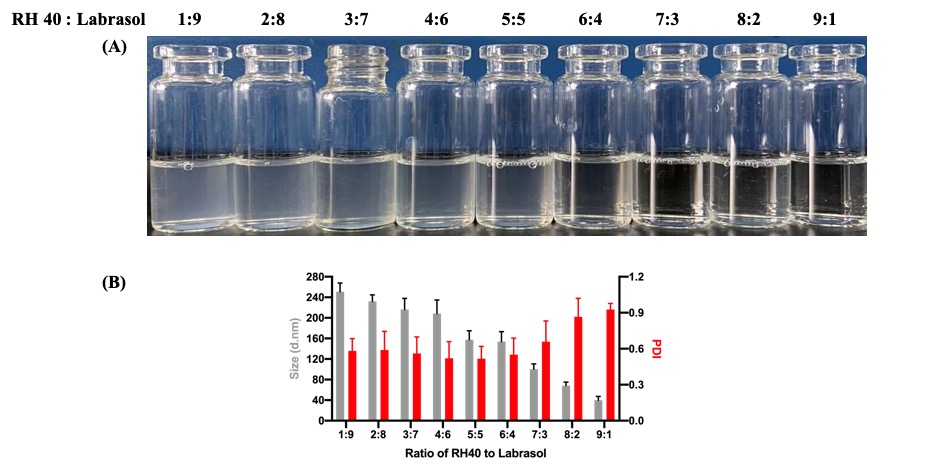


**Figure S3. Selection of the proper ratio of Labrasol and PEG-40 hydrogenated castor oil (RH 40) as surfactants.** (A) Emulsification efficiency of various ratios of Labrasol and RH 40 (37.5% in total) in combination with 1,2-propylene glycol (12.5%) when glyceryl triacetate was used as oily phase (50%). (B)The globule size and polydispersity index (PDI) of the corresponding formulations in (A). Data are expressed as mean ± SD (n = 3).


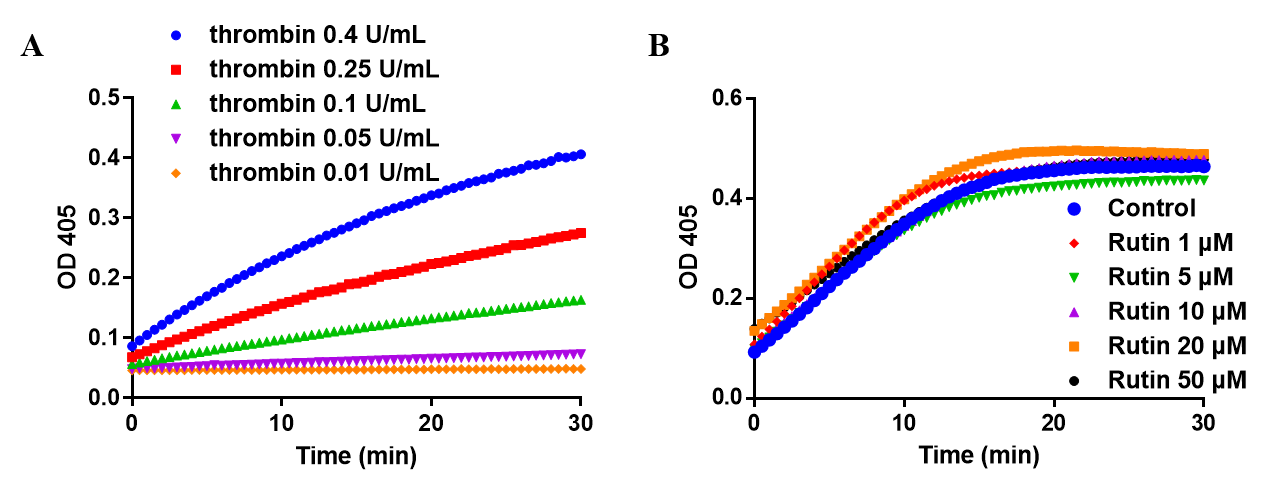


**Figure S4. Rutin did not inhibit thrombin proteolytic activity.** (A) The hydrolysis of chromogenic substrate S2238 by different concentrations of thrombin was determined. (B) The effect of different concentrations of rutin on the hydrolysis of S2238 by 0.4 U/mL of thrombin.

**Table S1. The composition, visual appearance, globule size and polydispersity index (PDI) of various formulations (mean ± SD, n = 3).**

| F | Glyceryl triacetate (%) | 1,2-propylene glycol (%) | | Labrasol (%) | | RH 40 (%) | | Appearance | Size (nm) | PDI |
| --- | --- | --- | --- | --- | --- | --- | --- | --- | --- | --- |
| 1 | 10 | | 22.5 | | 33.8 | | 33.8 | bluish | 115.5±9.2 | 0.34±0.10 |
| 2 |  |  | 30 | | 30 | | 30 | transparent | 40.5±3.4 | 0.11±0.04 |
| 3 |  |  | 45 | | 22.5 | | 22.5 | bluish | 77.6±8.3 | 0.41±0.09 |
| 4 | 15 | | 21.2 | | 31.9 | | 31.9 | bluish | 130.2±11.2 | 0.44±0.12 |
| 5 |  |  | 28.3 | | 28.3 | | 28.3 | transparent | 48.3±5.7 | 0.15±0.08 |
| 6 |  |  | 42 | | 21.5 | | 21.5 | bluish | 152.6±10.9 | 0.49±0.17 |
| 7 | 20 | | 20 | | 30 | | 30 | transparent | 44.6±4.7 | 0.15±0.06 |
| 8 |  |  | 26.7 | | 26.7 | | 26.7 | bluish | 133.9±12.4 | 0.51±0.15 |
| 9 |  |  | 40 | | 20 | | 20 | bluish | 150.4±14.3 | 0.29±0.09 |
| 10 | 30 | | 17.5 | | 26.2 | | 26.2 | bluish | 146.8±8.9 | 0.43±0.11 |
| 11 |  |  | 23.3 | | 23.3 | | 23.3 | bluish | 151.9±12.3 | 0.40±0.16 |
| 12 |  |  | 35 | | 17.5 | | 17.5 | bluish | 168.5±14.3 | 0.41±0.13 |
